# Long tails, short telomeres: Dyskeratosis congenita

**DOI:** 10.18632/oncotarget.4388

**Published:** 2015-06-09

**Authors:** Hemanth Tummala, Amanda J. Walne

**Affiliations:** Centre for Genomics and Child health, Blizard Institute, Barts and The London School of Medicine and Dentistry, Queen Mary University of London, London, UK

Dyskeratosis congenita (DC) is an inherited life threatening bone marrow failure syndrome [1]. The name dyskeratosis comes from the classic triad of clinical features nail dystrophy, abnormal skin pigmentation and leucoplakia in patients with DC. However the mortality in DC is primarily due to bone marrow failure and other complications such as pulmonary fibrosis, liver cirrhosis and early onset of cancer. DC is genetically heterogeneous and harbors constitutional mutations in key genes *(DKC1*, *TERC*, *TERT*, *NOP10*, *NHP2*, *TINF2*, *TCAB1, CTC1, RTEL1* and *ACD)* that are involved in telomere maintenance. In short, telomere maintenance is dependent on the catalytic function of telomerase (TERC-RNA component of telomerase and TERT-telomerase reverse transcriptase), telomerase stability (DKC1-dyskerin, NOP10-nucleolar protein 10 homolog and NHP2-non histone ribonucleoprotein 2 homolog), telomerase recruitment (TINF2-TRF1-interacting nuclear protein 2 and ACD-adeno cortical dysplasia homologue aka TPP1), telomerase trafficking (TCAB1-telomerase cajal body 1), telomerase docking (CTC1-conserved telomere maintenance component 1) and telomere replication (RTEL1-regulator of telomere length 1). DC patients carrying mutations in these genes possess critically short telomeres and an abnormal DNA damage response, causing selective exhaustion of cells in tissues with rapid turnover such as the bone marrow [1]. Challenges remain to understand the diverse spectrum of disease aetiology in DC. In addition the genetic characterisation of clinical cases to date accounts only about two thirds of the families in our DC registry in London.

In our latest work published in *The Journal of Clinical Investigation*, we describe the first constitutional biallelic mutations in the poly(A)-specific ribonuclease (*PARN*) gene in a subgroup of patients with severe DC [2]. These patients have very short telomeres but there had been no previous connection reported between PARN and telomere biology. PARN is an essential deadenylase enzyme that is involved in the control of mRNA stability and turnover. Specifically it trims poly(A) tails of mRNA to exert translational silencing for a large number of genes/transcripts. This is considered to be an important function in the regulation of several key cellular pathways including nonsense mediated RNA decay (NMD) and the DNA damage response. In our study we identified PARN deficiency, abnormal p53 regulation, G2/M phase cell cycle arrest and cell death upon DNA damage in patient cells harboring biallelic *PARN* mutations. PARN targets a discrete set of mRNAs for degradation in non-stress conditions, which includes p53 [3]. Upon DNA damage PARN binds to the accumulated p53 and other tumour suppressor proteins Cstf(50) – cleavage stimulation factor and BARD1-BRCA1 associated ring domain protein 1) to regulate nuclear deadenylation. Hence PARN activity is tightly controlled under different cellular conditions. What we have shown [2] is that PARN deficient patient cells clearly exhibited a decrease in the stability of several key telomere maintenance genes (*DKC1*, *TERF1*, *RTEL1* and *TERC*). However the question still remains as to how PARN deficiency specifically leads to telomere attrition in these patients with severe DC.

A recent study published in Nature Genetics reported heterozygous mutations in *PARN* associated with telomere attrition in patients with idiopathic pulmonary fibrosis [5]. This independent study, based on a different cohort of patients, also indicates a role for PARN in telomere maintenance. The authors of this study speculated that haploinsufficiency of PARN may cause telomere shortening through down regulation of human *TERC* [4]. However lessons from PARN deadenylase targets may imply a direct role in telomere maintenance [5]. For example, PARN trims oligoadenylated tails of H/ACA-box snoRNAs [5]. As *TERC* being an H/ACA-box snoRNA, and known to be oligoadenylated in a small fraction of the total *TERC* pool in human cells [6], it is therefore conceivable that PARN deficiency could result in accumulation of immature oligoadenylated *TERC* fraction. It is also notable that *TERC* associates with dyskerin (encoded by *DKC1*), and mutations in *DKC1* identified in DC patients exhibit low levels of *TERC*. We observed a decrease in *DKC1* transcript as well as dyskerin protein levels in patient cells with biallelic PARN mutations [2]. Furthermore, PARN knockdown studies in a heterologous cell system revealed a decrease in *DKC1* transcript stability after actinomycin D treatment. Hence reduction in *TERC* upon PARN deficiency could be a consequence of insufficient dyskerin. PARN deficiency could also impact on telomere maintenance via Cajal bodies, sites for telomerase trafficking and assembly, since depletion of PARN results in reduced size and number of Cajal bodies in human cells [5]. Finally a direct association of PARN to telomeres is also a possibility as it interacts with UPF1 – eukaryotic up-frameshift 1, a key NMD factor that localises to telomeres and regulates the polyadenylated telomeric repeat containing RNA (TERRA) [7].

In conclusion, it has been established that PARN deadenylation function is important in telomere biology. Irrespective of the precise mechanism(s), *PARN* mutations link the basic deadenylation pathway to telomeropathies such as idiopathic pulmonary fibrosis and dyskeratosis congenita.
